# A Novel Role for *Arabidopsis* CBL1 in Affecting Plant Responses to Glucose and Gibberellin during Germination and Seedling Development

**DOI:** 10.1371/journal.pone.0056412

**Published:** 2013-02-20

**Authors:** Zhi-Yong Li, Zhao-Shi Xu, Yang Chen, Guang-Yuan He, Guang-Xiao Yang, Ming Chen, Lian-Cheng Li, You-Zhi Ma

**Affiliations:** 1 Institute of Crop Science, Chinese Academy of Agricultural Sciences (CAAS)/National Key Facility for Crop Gene Resources and Genetic Improvement, Key Laboratory of Biology and Genetic Improvement of Triticeae Crops, Ministry of Agriculture, Beijing, China; 2 The Genetic Engineering International Cooperation Base of Chinese Ministry of Science and Technology, Chinese National Center of Plant Gene Research (Wuhan) HUST Part, College of Life Science and Technology, Huazhong University of Science & Technology (HUST), Wuhan, China; Instituto de Biología Molecular y Celular de Plantas, Spain

## Abstract

Glucose and phytohormones such as abscisic acid (ABA), ethylene, and gibberellin (GA) coordinately regulate germination and seedling development. However, there is still inadequate evidence to link their molecular roles in affecting plant responses. Calcium acts as a second messenger in a diverse range of signal transduction pathways. As calcium sensors unique to plants, calcineurin B-like (CBL) proteins are well known to modulate abiotic stress responses. In this study, it was found that *CBL1* was induced by glucose in *Arabidopsis*. Loss-of-function mutant *cbl1* exhibited hypersensitivity to glucose and paclobutrazol, a GA biosynthetic inhibitor. Several sugar-responsive and GA biosynthetic gene expressions were altered in the *cbl1* mutant. CBL1 protein physically interacted with AKINβ1, the regulatory β subunit of the SnRK1 complex which has a central role in sugar signaling. Our results indicate a novel role for CBL1 in modulating responses to glucose and GA signals.

## Introduction

Sugars modulate important processes during the almost all phases of plant development [1], [2]. High sugar concentration delays seed germination and inhibits cotyledon expansion and greening, true leaf formation and root growth of *Arabidopsis* seedlings [3]. Sugar also possesses a phytohormone-like function in regulating a series of genes that control a range of biological functions, such as photosynthesis, nitrogen metabolism and carbohydrate consumption [1], [4]. Sucrose, glucose and fructose are examples of sugars. Sucrose is stored in vacuoles or cleaved into glucose and fructose by invertases or UDP-glucose and fructose by sucrose synthases [5]. While sucrose is the main transported form of sugars in plants, glucose, as one of the more important sugars, has been shown to affect seed germination and early seedling development including cotyledon expansion and greening [6], [7]. Though fructose has long been proposed as a possible signaling molecule, fructose signaling in plants has remained largely unexplored [8]. During the past few decades, a number of *Arabidopsis* mutants involved in sugar signaling have been identified based on the negative effects of sugar on seed germination and post-germinative growth, and some of the genes are involved in abscisic acid (ABA) and ethylene signal pathways [9], [10]. For instance, the sugar insensitive mutant *sucrose uncoupled-6* (*sun6*) and glucose insensitive mutant *gin6* were found to be allelic to the mutant *abscisic acid insensitive-4* (*ABI4*), which is thought to be involved in ABA signal transduction [10]–[12]. The *sugar-insensitive1* (*sis1*) mutant is as allelic to *ctr1*, a previously identified mutant with a constitutive response to ethylene that displays multiple phenotypes of resistance to glucose and mannose as well as the gibberellin (GA) biosynthesis inhibitor paclobutrazol (PAC) [13]. These findings suggest extensive interactions between sugar and hormone signaling pathways and raise a possibility that genes involved in sugar-hormone cross talk might be transcriptionally regulated by sugar [7], [14]. And more studies have also provided significant evidence of interactions between sugar and phytohormone response as well as other metabolic pathways [7], [14]–[16]. However, because of the dual function of sugars as nutrients and signaling molecules in plants, sugar signaling transduction is still confused.

Ca^2+^ acts as a second messenger in many of the diverse range of signal transduction pathways in plants. Multiple extracellular signals, such as hormones and biotic and abiotic stimuli, elicit changes in Ca^2+^ levels in the cell [17], [18]. Specific Ca^2+^ signatures trigger a wide range of signal transduction pathways through various calcium sensors. All eukaryotic cells have multiple calcium sensors and many of them have been identified. Among them, the calcineurin B-like (CBL) proteins family is unique to plants. To date, they are generally known to be involved in abiotic stress responses. *Arabidopsis* CBL1 functions as a positive regulator in salt or drought stress responses [19], [20]. CBL9 affects plant responses to salt and mannitol as well as regulating osmotic stress-induced ABA accumulation in *Arabidopsis* [21]. The CBL protein family in rice is induced by various stress signals, and one of them, *OsCBL8*, improves salt tolerance in transgenic plants [22]. Maize *ZmCBL4* was shown to modulate salt stress-elicited calcium signaling and thus tolerance to salinity [23]. Although evidence is limited, previous reports have noted Ca^2+^ involvement in sugar signal transduction [24], [25]. Application of exogenous sugar induces accumulation of Ca^2+^ in the cell cytosol and increased Ca^2+^ may initiate Ca^2+^ signaling leading to the expression of sugar response genes, suggesting Ca^2+^ involvement in sugar signal transduction [25]. Global transcription profiling revealed that a number of genes associated with Ca^2+^ regulation including *CaBP-22*, annexin and several calcium ion binding proteins were induced by exogenous 3% glucose [15]. Sugar signalling cascade has been shown to involve mitogen-activated protein kinases, protein phosphatases, Ca^2+^ and calmodulin to result in various types of plant responses [26]. However, there is rare evidence to implicate a role CBLs in sugar signaling.

Here, we report the molecular and genetic characterization of CBL1 in a glucose signaling context, and its close interaction with GA signaling during seed germination and early seedling development. In this study, we found that the expression of *CBL1* was mainly induced by glucose, and alteration of glucose-response gene expression in *cbl1* mutant was consistent with a glucose hypersensitive phenotype. Subsequent research indicated that *cbl1* did not alter its response to ABA or ethylene, whereas it was hypersensitive to PAC. Moreover, in a yeast two-hybrid screen we identified an interactor named AKINβ1, which was previously reported to be involved in sugar signaling [27]. Taken together, we suggest a novel role for CBL1 in affecting plant responses to glucose and GA signals.

## Materials and Methods

### Plant Materials and Growth Conditions

Seeds from *Arabidopsis* (*Arabidopsis thaliana*), ecotype Col-0, were used as wild type in this study. Both seeds of *Arabidopsis* (Columbia, Col-0) and T-DNA insertion lines of *cbl1* were surface sterilized with 30% bleach for 10 min and extensively washed five times with sterile water. Sterile seeds were plated on ½ Murashige and Skoog (MS) medium plates in darkness for 3 d at 4°C, and then transferred to a growth chamber with a 16 h light period at 22°C. Screening the homozygous T-DNA insertion line (SALK_110426) was conducted by PCR with gene-specific primers SALK_110426LP: 5′ -GGGCTACGATACATTGAATCG- 3′; SALK_110426RP: 5′ -TTGATCGTCTGGTTTCGAATC- 3′ and T-DNA border primer LBb1.3∶5′ -ATTTTGCCGATTTCGGAAC- 3′. Homozygous mutant plants were further confirmed by RT-PCR with gene-specific primers of *CBL1* (GenBank accession number: AT4G17615): 5′ -AATGAAACTGGCTGATGAAACC- 3′ (forward) and 5′ -CCTCCGAATGGAAGACAAAACT- 3′ (reverse).

### RNA Extraction and Real-time PCR Analyses

Two-week-old *Arabidopsis* seedlings of wild-type (Col-0) plants were immersed in solutions containing 3% glucose, 3% sucrose or 3% fructose at room temperature for 6 h. Seedlings were collected and immediately frozen in liquid nitrogen. Total RNA was extracted from these plants using Trizol reagent (Takara) and treated with RNase-free DNase I (Takara). For real-time PCR, 2 µg of total RNA was used for first strand cDNA synthesis with a PrimeScript 1st Strand cDNA Synthesis kit (Takara). Quantitative expression assays were performed with the SYBR *Premix Ex Taq™* kit (Takara) and an ABI 7300 according to the manufacturer’s protocols (Applied Biosystem). The PCR program was 95°C for 2 min followed by 40 cycles of denaturation for 15 s at 95°C and annealing/extension at 60°C for 1 min. Expressions of all genes were assayed in triplicate. Gene expression was calculated with the Delta-Delta cycle threshold method [28]. Relative quantitative results were calculated by normalization to *UBQ10* (GenBank accession number: AT4G05320). The primer pairs used for real-time PCR are listed in Table S1.

### Glucose and Mannitol Responses Assays

For germination assays, seeds of Col-0 and *cbl1* mutants were surface sterilized and water imbibed in the dark for 3 d at 4°C. After stratification, 80 to 100 seeds of each genotype were sown in triplicate in petri dishes containing ½ MS medium, supplemented or not with 3% of glucose or 3% mannitol, before transfer to the growth chamber (16 h photoperiod). Germination (defined as the protrusion of the radicle through the seed coat), cotyledon greening, and cotyledon expansion were scored every day after transfer to the growth chamber, and cotyledon greening and expansion rates were calculated over the total of germinated seeds. For the root growth assay, the seven-day-old seedlings were placed vertically on ½ MS medium in the presence of zero, 3% glucose, or 3% mannitol. Root lengths were measured five days later. Average percentages were calculated with standard errors of the triplicates.

### Phytohormones and Paclobutrazol Responses Assays

For the ABA or PAC responses assays, the surface-sterilized seeds were placed on ½ MS plates, supplemented or not supplemented with a gradient of ABA or PAC concentrations (from 0.2 µM to 1 µM). Seeds were stratificated at 4°C for 3 d and germinated under a 16 h light photoperiod at 22°C. Germination, scored by radicle emergence from the seed coat, was recorded each day.

For assessment of hypocotyl elongation, 50 seeds of each genotype were surface sterilized and stratified as described above, plated in ½ MS plates supplemented with zero or appropriate concentrations of ethylene precursor aminocyclopropane carboxylic acid (ACC), and grown vertically in complete darkness at 22°C for five days. Hypocotyl lengths were measured using IMAGEJ software (http://rsbweb.nih.gov/ij). All assays were repeated at least three times with similar results.

### Genes Isolation and Vectors Construction

The full coding sequence of *CBL1* was amplified with two primers (5′ -ACTGGATCCATGGGCTGCTTCC- 3′; *Bam*HI site underlined) and (5′ -CTAGTCGACTGTGGCAATCTCATC- 3′; *Sal*I site underlined). For yeast two-hybrid assays, the PCR product was cloned into the pGBKT7 vector (Clontech) to generate the pGBKT-CBL1 bait vector. The full coding sequence of *AKINβ1* (GenBank accession number: AT5G21170) was amplified with two primers (5′ -CATGGATCCATGGGAAATGCG- 3′; *Bam*HI site underlined, and 5′ -TGACTCGAGCCGTGTGAGCGGTT- 3′; *Xho*I site underlined) and cloned into the prey vector pGADT7-Rec2 (Clontech) to generate a pGADT7-AKINβ1 vector. For the GST pull-down assay, the PCR product of CBL1 was cloned into the pGEX-4T-1 vector (Amersham) as pGEX-CBL1 in-frame to the coding sequence of glutathione S-transferase (GST), and the PCR product of AKINβ1 was cloned into pET-28a(+) vector (Novagen) to generate pET-AKINβ1 containing a His-AKINβ1 fusion construct under control of a T7 bacterial expression promoter. All constructs were confirmed by sequencing.

### Yeast Two-hybrid Screening

The *Arabidopsis* seedling cDNA library was constructed in a pGADT7-Rec2 vector containing a GAL4 activation domain using Matchmaker Library Construction (Clontech) and then transformed into the yeast strain AH109 (Clontech). The bait vector pGBKT-CBL1 was transformed into yeast strain Y187 (Clontech). Yeast two-hybrid screening was performed using the MATCHMAKER two-hybrid system (Clontech). After the transformed library was induced by polyethylene glycol, cells were plated on a synthetic dropout (SD) medium that lacked Trp, Leu, Ade and His (SD-Trp-Leu-Ade-His), but was supplemented with an optimal concentration of 10 mM 3-amino-1,2,4-triazole (3-AT) to reduce any artificial interaction. Selected clones were sequenced by a T7 primer. The full sequences of the candidates were cloned from *Arabidopsis* cDNA and used for vector construction. These vectors were retransformed with the bait vector into the yeast strain AH109 for two-hybrid analysis. Transformants were selected by growing on SD-Trp-Leu- at 30°C for 4 d. Surviving clones were retransferred to SD-Trp-Leu-His-Ade- medium and assayed for β-galactosidase activity according to the manufacturer’s instructions (Clontech).

### Protein Expression and Pull-down Assay

The recombinant vectors pGEX-CBL1 and pET-AKINβ1 were expressed in *Escherichia coli* strain BL21 (DE3) by induction with 0.5 mM isopropyl-1-thio-b-D-thiogalactoside (IPTG) at 22°C and purified with Glutathione Sepharose or Ni-activated His-binding resin (Amersham) according to the manufacturers’ instructions. Approximately 5 µg of GST-CBL1 fusion protein was immobilized on Glutathione-Sepharose 4B beads and incubated with His-tagged AKINβ1 proteins. After incubation overnight at 4°C on a rotary incubator, the beads were washed five times with ice-cold phosphate-buffered saline, re-suspended in SDS gel-loading buffer, and loaded on SDS-PAGE (10%, w/v) gels. Protein bound to GST-CBL1 was detected by Western blotting with anti-His antibody (Amersham) and visualized using chemiluminescence following the manufacturer’s recommendations (Amersham).

### Bimolecular Fluorescence Complementation (BiFC) Assay

For BiFC analysis, the full-length coding sequence of CBL1 was cloned into *BamH*I and *Xho*I sites in a PUC-pSPYNE vector [29] and fused with the N-terminal fragment of YFP to form a YFPN-CBL1 construct. The full-length coding sequence of AKINβ1 was cloned into the same restricted sites in a PUC-pSPYCE vector [29] as a fusion with the C-terminal fragment of YFP to form a YFPC-AKINβ1 construct. For transient expression, plasmids of YFPN-CBL1 and YFPC-AKINβ1 were co-transformed into *Arabidopsis* protoplasts following a previously described protocol [30]. Fluorescence in protoplasts was visualized by a confocal laser scanning microscope (Leica Microsystem, Heidelberg, Germany) 16 h after incubation at room temperature.

## Results

### *CBL1* is Induced by Sugar and *cbl1* Mutant Plants are Hypersensitive to Glucose

**Figure 1:**
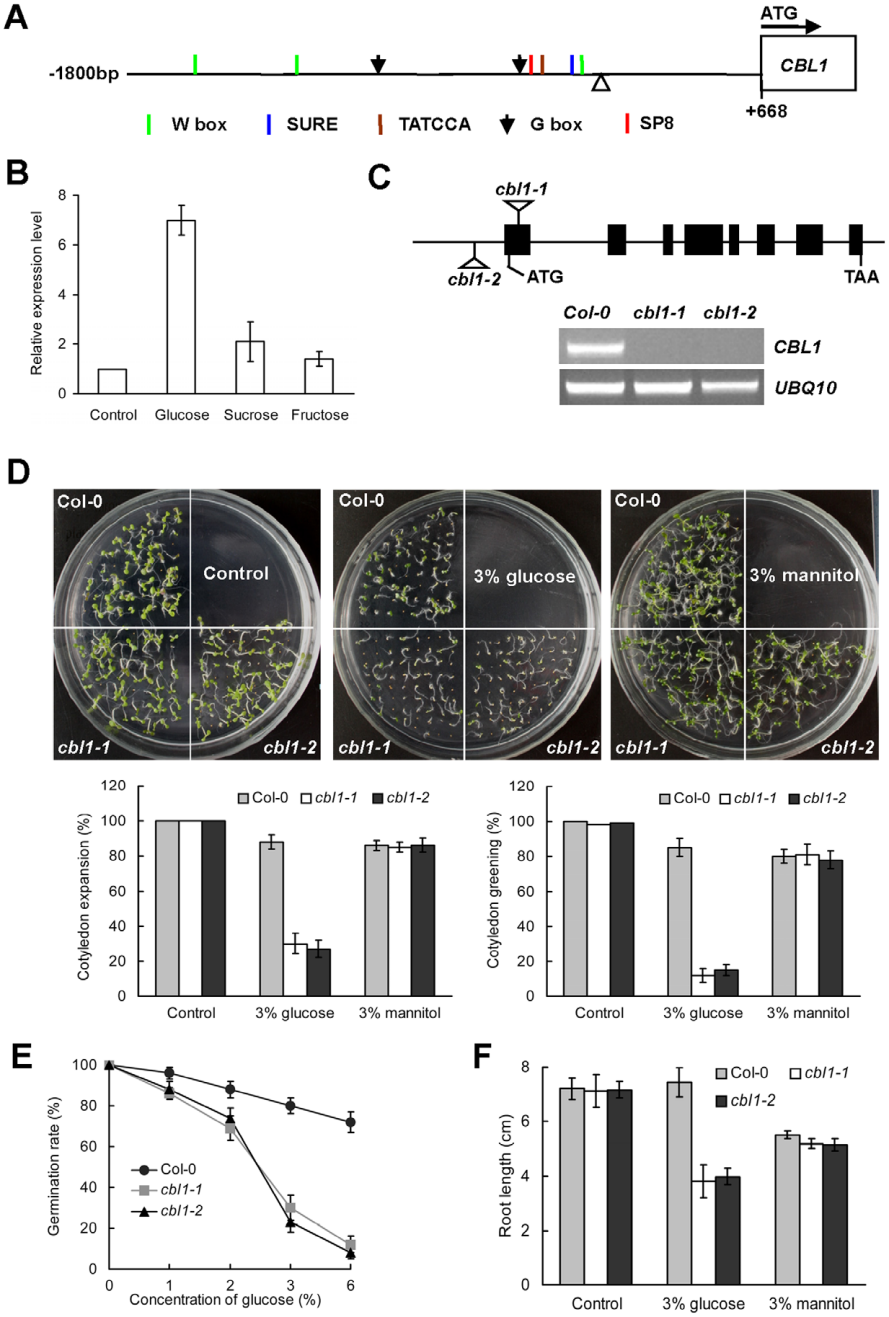
Expression patterns of *CBL1* and glucose sensitivity of *cbl1* mutant. (A) Deduced *cis*-acting elements present in the promoter region of *CBL1*. (B) *CBL1* was induced by sugars. Seven-day-old Col-0 seedlings were transferred to liquid media containing 3% glucose, 3% sucrose and 3% fructose. Plants immersed in water without sugar served as a control. (C) Isolation of *cbl1* T-DNA insertional mutant. T-DNA insertion sites were at 75 bp downstream and 265 bp upstream of ATG, respectively. Black boxes and solid lines denote exons and introns, respectively. No *CBL1* transcript was detected by RT-PCR in mutant plants. (D) Representative growth phenotypes of Col-0 and *cbl1* seeds grown in zero, 3% glucose or 3% mannitol for 7 days. Bars = 3 mm. Germination and early seedling development (cotyledon greening and expansion) rates, scored 7 d after stratification, of Col-0 and *cbl1* mutant seedlings grown in control conditions or in the presence of 3% glucose or 3% mannitol (means ± SE, n = 3). (E) Germination rates of Col-0 and the mutant in the presence of different concentrations of glucose. Each point represents averages of 80 seeds ± SD. (F) Effects of glucose and mannitol on root elongation of Col-0 and mutant seedlings. Seven-day-old seedlings were transferred from regular ½ MS medium to media containing zero, 3% glucose, or 3% mannitol. Root lengths were measured 5 days after transfer. Data represent averages of 30 plants ± SD.

*Arabidopsis* CBL9 loss-of-function line *cbl9* is hypersensitive to glucose [21]. CBL1 may share some overlapping functions with CBL9 due to the high similarity (∼89%) in their coding regions [31]–[33]. Thus, whether CBL1 is involved in sugar response was remained to be examined. Analysis of 1.8 kb sequence upstream of the transcriptional start site of the *CBL1* promoter [19], [20] using the PLACE (www.dna.affrc.go.jp/PLACE) and PlantCARE (http://bioinformatics.psb.ugent.be/webtools/plantcare/html/) databases showed that several potential *cis*-acting elements related to sugar response were present, such as W box, sugar response element (*SURE*), TATCCA element, and SP8 motif [34]–[37] (Fig. 1A and Figure S1), further implying a possible role of CBL1 in sugar response. Next we tested the expression of *CBL1* in response to sugars. The expression pattern showed that *CBL1* was obviously induced by glucose, whereas no significant increased *CBL1* mRNA levels could be observed following exposure of the plants to 3% sucrose or 3% fructose (Fig. 1B). The results indicate that the expression of *CBL1* was specifically induced by glucose.

To further investigate the function of CBL1 in sugar response, seed germination and seedling development of *CBL1* T-DNA insertion mutants were evaluated. The two *cbl1* mutant had a different T-DNA insertion site in the genome sequence of *CBL1*, respectively. In *cbl1-1*, the T-DNA inserted in the first exon of the gene, and in *cbl1-2*, the T-DNA inserted in the 5′-untranslated region as previously described [20], [38], and both of them did not produce a detectable amount of *CBL1* transcript in a reverse transcription experiment (Fig. 1C). In the absence of glucose, there were no obvious morphological or developmental differences between wild-type (Col-0) and *cbl1* plants (Fig. 1D, Control). Though the exogenous supply of 3% glucose had no obvious effect on Col-0 plants, both the expansion and greening of mutants’ cotyledons were significantly affected by the presence of glucose with *cbl1* seedlings only being able to expand around 30% and green about 10% of their cotyledons (Fig. 1D). In contrast to glucose, the effect of equimolar concentrations of mannitol was similar on Col-0 and mutants for the two parameters. These findings revealed that *CBL1* disruption led to glucose hypersensitive in *Arabidopsis* seedling development. In the presence of glucose, germination in *cbl1* mutant lines was also more sensitive to glucose than that in Col-0. For example, the germination rate decreased to 83% for Col-0 plants and about 30% for *cbl1* after stratification for 7 d in the presence of 3% glucose (Fig. 1E). This phenomenon was even more serious in mutant lines with the increase of glucose concentrations, suggesting that disruption of *CBL1* in *Arabidopsis* enhances the sensitivity to glucose during seed germination. Moreover, root elongations were rarely arrested in Col-0 after treatment with 3% glucose for 5 d, whereas glucose clearly inhibited root growth in *cbl1* mutant plants (Fig. 1F and Figure S2). These results showed that repression of CBL1 increased the inhibitory effects of glucose on germination and seedling growth.

### Glucose-responsive Genes Expression is Altered in *cbl1* Mutant Plants

**Figure 2:**
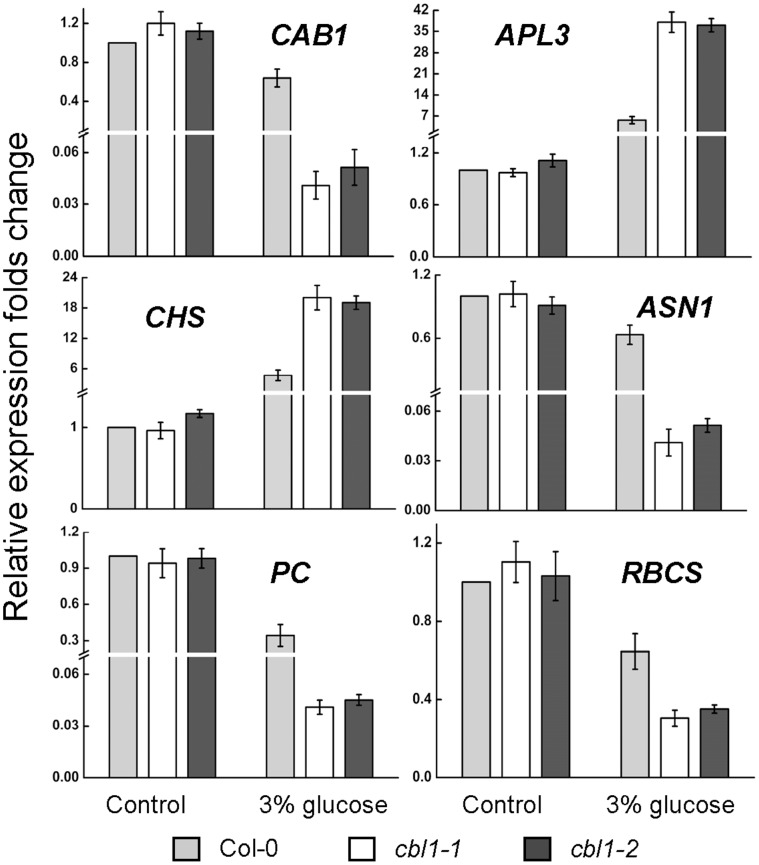
Real-time PCR analysis of expression of glucose-responsive genes in Col-0 and *cbl1* mutant plants treated with zero or 3% glucose. Mean values were normalized to the transcript level of the internal control, *UBQ10*. *CAB1*, *chlorophyll a/b-binding protein 1*; *PC*, *plastocyanin*; *ASN1*, *asparagine synthetase 1*; *CHS*, *chalcone synthase*; *APL3*, *ADP-glucose pyrophosphrylase*, large subunit; *RBCS*, *ribulose-1,5-bisphosphate carboxylase*, small subunit.

It has been shown that high levels of sugar reduce transcription of genes for photosynthesis and nitrogen metabolism while promoting expression of genes related to carbohydrate consumption and storage [7], [39]. To further examine the involvement of CBL1 in the glucose response, the expression levels of several glucose-response genes were analyzed by real-time PCR in Col-0 and *cbl1* mutant in the presence or absence of glucose (Fig. 2). Among these genes, three were photosynthetic genes: *CAB1* (AT1G29930) encoding a chlorophyll a/b-binding protein, the nuclear-encoded photosynthesis gene *plastocyanin* (*PC*) (AT1G76100) and *RBCS*, encoding the small subunit of *ribulose-1,5-bisphosphate carboxylase* (AT5G38410). In the absence of glucose, these genes were expressed similarly among the plants. In Col-0 seedlings 3% glucose was insufficient to repress *CAB1*, whereas *PC* and *RBCS* transcript levels were reduced about 2∼3 folds. In the *cbl1* mutants, by contrast, down-regulation of the expression of these genes by 3% glucose was much more pronounced, with both transcripts of *CAB1* and *PC* being highly reduced. Similarly, 3% glucose exerted only a slight repression on the nitrogen metabolism gene asparagine synthetase 1 (*ASN1*) (AT3G47340) in Col-0 seedlings but strongly repressed its expression in the *cbl1* mutant. In contrast, 3% glucose promoted expression of *ADP-Glucose pyrophosphrylase*, large subunit (*APL3*) (AT4G39210), a rate limiting enzyme for starch synthesis and the anthocyanin biosynthesis gene *chalcone synthase* (*CHS*) (AT5G13930) for several folds in Col-0. And expression of the two genes by 3% glucose was much more pronounced with dozens of folds in mutant plants. These results are consistent with *cbl1* mutant having a glucose-sensitive phenotype.

### Suppression of *CBL1* Increases Sensitivity to PAC

**Figure 3:**
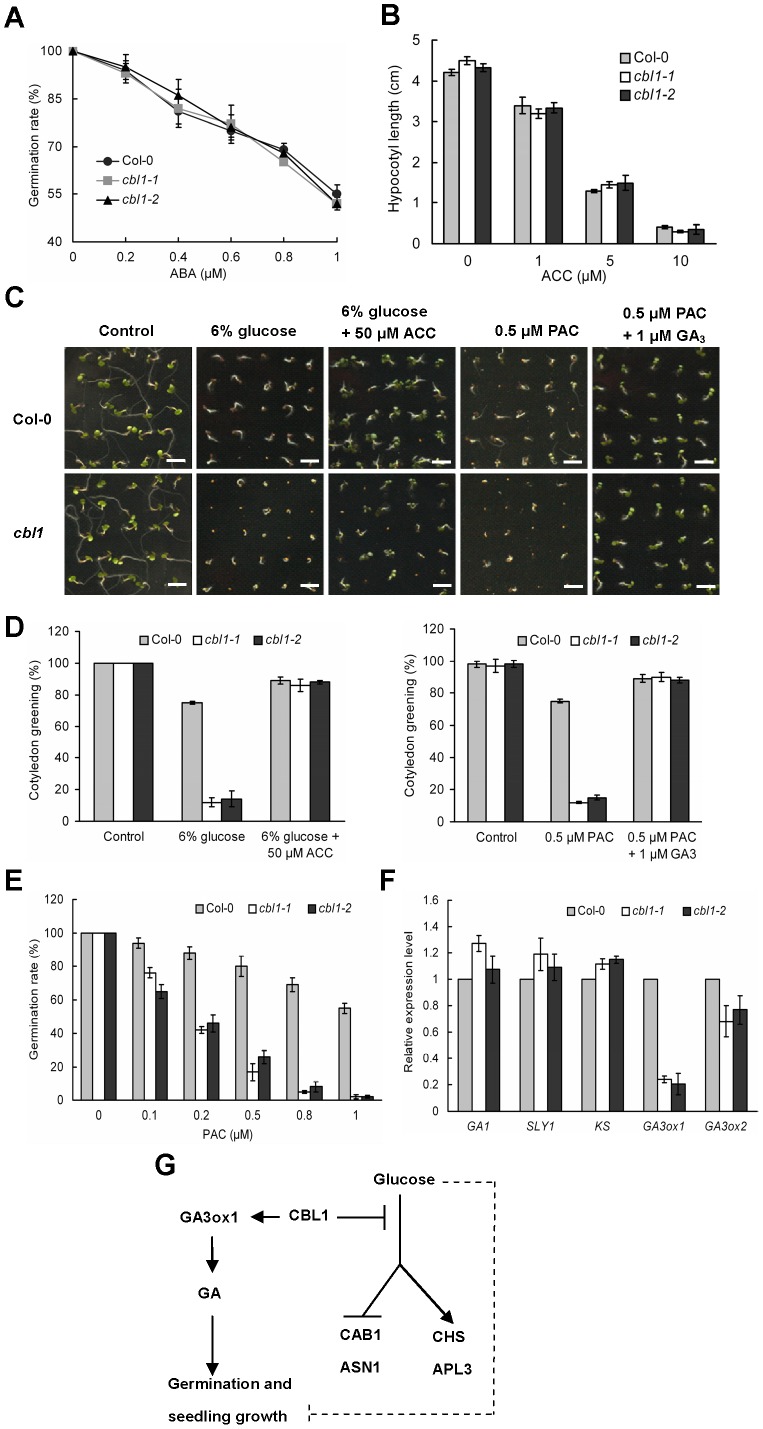
Phytohormones responses in *cbl1* mutant plants. (A) Germination rates of Col-0 and mutant seeds after 5 d of incubation at 22 °C on ½ MS medium containing different concentrations of ABA. Results are presented as average values ± SE from three experiments. (B) Effect of ACC on hypocotyls elongation in Col-0 and mutant. Hypocotyl lengths were measured 5 d after stratification of seedlings grown in the dark in the presence of different concentrations of ACC. (C) Representative images of Col-0 and mutant grown in conditions indicated above the images for 7 days. Bars = 5 mm. (D) Comparison of percentage green cotyledons of Col-0 and mutant seedlings grown in control conditions or in the presence of 6% glucose supplemented or not with 50 µM ACC or in the presence of 0.5 µM PAC supplemented or not with 1 µM GA_3_. Data are averages of 60 plants ± SD. (E) Germination of Col-0 and mutant grown in medium with different concentrations of PAC. Each point represents averages of 80 seeds ± SD. (F) Relative expressions of GA biosynthetic and signaling genes in two-week-old seedlings of Col-0 and mutant grown on MS medium. *G1D1*, *GA INSENSITIVE DWARF1A*; *SLY1*, *SLEEP1*; *KS*, *ent-kaurene synthase*; *GA3ox*: *GA3-oxidase*. (G) A model to explain CBL1 function in glucose- and GA-responsive gene expression. CBL1 might positively modulate GA response via regulation of *GA3ox1*, and affects both glucose-induced and glucose-repressed gene expression.

Previous studies demonstrated vital roles for ABA and ethylene in plant glucose responses [10], [40]. It was therefore important to investigate the effect of the *cbl1* mutation on ABA and ethylene signaling. A germination assay on medium containing ABA was conducted to investigate whether the glucose phenotype conferred by the *CBL1* mutation is accompanied with altered sensitivity to ABA, as appears to be the case for the vast majority of the identified sugar response mutants. As shown in Fig. 3A, the exogenous application of ABA delayed germination of Col-0 and *cbl1* seeds to a similar extent. This indicated that hypersensitivity to glucose in *cbl1* mutant is not due to ABA sensitivity. In order to determine the response of the *cbl1* mutant to ethylene, hypocotyl lengths of seedlings grown in the dark in the presence of ACC were measured on the basis that dark-grown seedlings undergo morphological modifications in the presence of ethylene. This is referred to as the ethylene triple response and includes shortening of the hypocotyls. As shown in Fig. 3B, the *cbl1* mutation also did not alter sensitivity to ethylene precursor ACC, with the hypocotyl length decreasing to similar extents in both Col-0 and the mutant. Ethylene is known to antagonize glucose response [9]. As expected, the addition of 50 µM ACC led to a recovery in cotyledon greening in *cbl1* mutant plants under high glucose concentration (Fig. 3C and D). These experiments indicated that *cbl1* was not defective in responding to ABA and ethylene signaling.

GA promotes seed germination and plant growth. Further study was undertaken to investigate whether *CBL1* supression will affect plant response to GA. The results showed that *cbl1* mutant was hypersensitive to PAC. A sharp decline in germination percentage of the *cbl1* mutant was observed in the presence of different concentrations of PAC (Fig. 3E). For instance, in the presence of 0.5 µM PAC, seed germination of the mutant was very low with around 20% germination rate, whereas about 78% of Col-0 germinated normally. This phenomenon was even more serious in mutant lines with the increase of PAC concentrations. When 0.5 µM PAC and 1 µM GA_3_ were supplied together, germination and cotyledon greenig of the mutant was largely restored (Fig. 3C and D). We supposed that hypersensitivity to PAC in *cbl1* mutant plants could be due to GA-deficiency. Analysis of genes expression for GA biosynthetic and signal pathway in both Col-0 and *cbl1* mutant plants was performed. Most genes selected for assay were not obviously changed in the *cbl1* mutant compared to Col-0. However, expression of the key GA biosynthetic gene *GA3ox1* was clearly reduced in *cbl1* plants (Fig. 3F). These results suggest that CBL1 probably affects the GA response by regulating the expression of *GA3ox1*.

### Identification of CBL1-interacting Proteins

**Figure 4:**
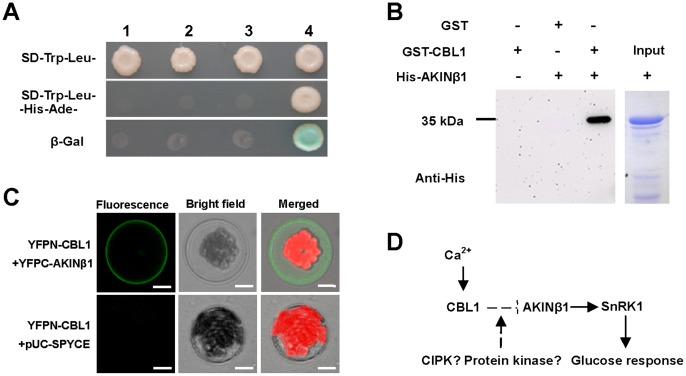
Interaction between CBL1 and AKINβ1. (A) Yeast two-hybrid interactions. Vectors were co-introduced into yeast AH109 strain in different combinations: 1, pGADT7 and pGBKT7; 2, pGADT7-AKINβ1 and pGBKT7; 3, pGADT7 and pGBKT7-CBL1; 4, pGADT7-AKINβ1 and pGBKT7-CBL1. Transformants were placed on selection medium and grown for 4 d before the β-galactosidase (β-Gal) assay. (B) *In vitro* GST pull-down assay. GST-CBL1 and His-AKINβ1 were expressed in *E. coli* and used for analysis. The presence or absence of each protein in the reaction mixture is shown as+or -, respectively. The right row shows a Coomassie Brilliant Blue-stained SDS-PAGE gel indicating captured proteins used as controls. Experiments were performed 3 times and a representative result was shown. (C) *In vivo* BiFC assay. The plasmids YFPN-CBL1 and YFPC-AKINβ1 were co-transformed into *Arabidopsis* protoplasts. The expression of CBL1 alone (YFPN-CBL1+ pUC-SPYCE) was used as control. Photographs were taken with a confocal laser-scanning microscope (Leika TCS-NT). Bars = 10 µm. (D) A proposed model to illustrate biological relevance of the interaction between CBL1 and AKINβ1. In the presence of calcium, CBL1 together with certain CBL-interacting protein kinases (not only CIPK) forms a complex to modify AKINβ1, thus affecting the activity of SnRK1. Positive interaction is noted by an arrow and bars indicate repression.

CBL1 is known to be associated with a number of proteins in exerting its functional biological roles. By using the yeast two-hybrid system, a positive interactor of CBL1 was identified and sequence analysis revealed that the CBL1-interacting protein was the β regulatory subunit of the heterotrimeric complex of sucrose non-fermenting-1-related protein kinase 1 (SnRK1) named AKINβ1. In yeast two-hybrid screening, strong growth on SD-Trp-Leu-Ade-His medium and activity of the reporter gene were observed only in yeast cells co-transformed with pGBKT7-CBL1 and pGADT7-AKINβ1 (Fig. 4A), indicating interaction of CBL1 and AKINβ1 in yeast. The interaction between CBL1 and AKINβ1 was further tested using in vitro pull-down assay with His-AKINβ1 and glutathione S-transferase (GST)-CBL1. As shown in Figure 4B, His-AKINβ1 also physically interacted with GST-CBL1 as shown by anti-His antibody on the resulting western blot. Moreover, the *in vivo* BiFC assay showed that the interaction of CBL1 and AKINβ1 occurred on the plasma membranes of *Arabidopsis* protoplasts (Fig. 4C). These results demonstrate that AKINβ1 interacts with CBL1.

## Discussion

### CBL1 is Involved in Sugar Response

As the important energy sources and structural storage components, sugars act to influence plant development. Also, there are increasing evidences that sugar plays important roles as a signaling molecule. For instance, glucose plays crucial roles in plant development both as an energy source and a signaling molecule, which is further associated with phytohormones [3], [7]. However, factors that affect glucose and phytohormone responses remain to be fully elucidated.

*Cis*-elements are important molecular switches involved in the transcriptional regulation of a dynamic network of gene activities controlling various biological processes. Though there is no common cis-element as a marker for the sugar signaling pathway, *cis*-elements involved in sugar signaling have been reported [3]. The activation of gene expression by sugars has been best studied with the promoters of genes encoding patatin, amylase, and vegetative storage protein [36], [41]–[44]. For instance, analysis of a short-inducible promoter derived from *β-Amy* indicated that TGGACGG element plays an important role in sugar inducible expression of both of the truncated promoters of *spo* and *β-Amy* [43]. A number of various sucrose response (repression or induction) elements (SURE) from several nuclear genes, including *rbcS* and *SPF1*, have been identified [37], [44]. The investigation of rice *α*-amylase gene reveals four essential *cis*-elements that are important for high sugar starvation-induced expression: the GC-box, the G-box, the SP8 motif, and the TATCCA element [34], [45]. A WRKY-type SUSIBA2 is sugar inducible and binds to the SURE and W-box [35]. The core sequence of the WRKY binding element (W-box) is found in the promoters of wheat, barley, and wild oat *α*-AMY2 gene [46]. As shown in Fig. 1A and Figure S1, several potential *cis*-acting elements respond to sugar signal, such as W-box, TATCCA element, WRKY-type SUSIBA2 and SP8 motif were present in the putative promoter of *CBL1*; suggesting CBL1 is sugar inducible gene. In *Arabidopsis*, CBL1 shares highest identity with CBL9 at the level of amino acid sequence among the nine CBLs [31]–[33]. Regarding the similarity in the amino acid sequence, expression pattern, and interactive CIPKs, we speculate that that CBL1 and CBL9 function in similar processes and share common functions [19]–[21], [31]–[33], [47]. Indeed, both *CBL1* and *CBL9* are activated by various abiotic stresses in *Arabidopsis* [19]–[21]. Particularly, using loss-of-function showed that the *CBL9* mutant was hypersensitive to glucose [21]. Thus, these findings inspired us to investigate whether CBL1 is involved in sugar response.

Next we tested the expression of *CBL1* in response to sugars by using real-time PCR assays. Here we found that *CBL1* was obviously induced by glucose (Fig. 1B). Further study showed that the loss-of-function *cbl1* mutant was hypersensitive to exogenous glucose. With the steady increase of glucose concentrations (0%–3%), seed germination and seedling development were arrested in both Col-0 and *cbl1* mutant lines, but more seriously inhibited in *cbl1* seedlings (Fig. 1D, E and F). Although *cbl1* mutant has displayed an osmo-sensitive phenotype [19], [20], this osmo-sensitive phenotype is insufficient to explain its glucose-sensitive phenotype. This conclusion is based on the finding that a developmental difference between Col-0 and mutant plants was not observed in germination and seedling growth when both genotypes were grown in the presence of 3% mannitol, which is enough to exert osmotic stress (Fig. 1D and F). This is consistent with previous studies that intermediate glucose concentrations (1.5%–3%) dramatically delay WT seed germination, similar concentrations of mannitol or sorbitol have very little effect, indicating that the effect of glucose is not simply osmotic [6], [48]. In addition, no significant difference between Col-0 and *cbl1* mutant plants was observed in the presence of equimolar sucrose (data not shown). In contrast, loss-of-function *cbl1* mutant was hypersensitive to 3% glucose (Fig. 1E and F), indicating that germination and growth arrest is glucose-specific and not due to osmotic stress. Moreover, *cbl1* mutant displayed a glucose hypersensitivity phenotype at the molecular level in regulating glucose-responsive gene expression (Fig. 2). Elevated sugar levels have long been known to down-regulate the transcription of both photosynthetic genes and genes associated with nitrogen metabolism, while upregulating the expression of genes involved in the synthesis of polysaccharides [4]. Following treatment with 3% glucose, several genes involved in photosynthesis including *CAB1*, *PC* and *RBCS* as well as *ASN1* for nitrogen metabolism were decreased in Col-0 [49], [50], but the decreases were more pronounced in *cbl1* mutant seedlings (Fig. 2). Sugar-induced anthocyanin accumulation is a common phenomenon in plant species and a sugar induction of anthocyanin biosynthesis was also demonstrated in *Arabidopsis* seedlings [51], [52]. ADP-glucose pyrophoshorylase (ADP-Glc PPase) catalyzes the first and limiting step in starch biosynthesis, which is composed of two types of subunits (small and large) [53], [54]. In plants, only the large subunit of *APL3* and *APL4* were sugar induced [55]. As expected, real-time PCR analysis showed that transcript levels of *CHS* associated with anthocyanin biosynthesis and *APL3* were induced by glucose in Col-0. In the *cbl1* mutant, induction of the expression of these genes by 3% glucose was much more pronounced, with both transcripts being highly overinduced. Thus, the *CBL1* mutation caused expression of all sugar-responsive genes to be more sensitive to exogenous glucose, suggesting that CBL1 may negatively regulate sugar-responsive gene expression by affecting a common part of the signaling pathways for both sugar-induced and sugar-repressed gene expression (Fig. 3G). Previous studies showed that repression of photosynthesis-related gene expression, such as *CAB1*, *PC*, and *RBCS*, was correlated with a hexokinase (HXK1)-mediated signaling function, whereas the effect of glucose on expression of *CHS* and *ASN1* was independent of HXK1-mediated signaling [56], [57]. These results suggest that CBL1 might be involved in both HXK1-dependent and HXK1-independent pathways.

### CBL1 Physically Interacts with AKINβ1 and Probably Opposes its Function in Sugar Signaling

Ca^2+^ acts as a second messenger in many of the diverse signal transduction pathways in plants. Calcium sensors, such as calmodulin (CaM), calmodulin domain protein kinases (CDPK), and CBLs, bind Ca^2+^ and change their conformation [31]–[33], [58]–[60]. CDPKs are protein kinases, whereas CaM and CBL are small Ca^2+^ sensors that do not have apparent enzymatic activities. Studies have shown that CaMs associate with a number of diverse target proteins, such as NAD kinase, glucose decarboxylase and transcription factors [59]. The well known CBL-interacting proteins are a group of sucrose non-fermenting-related serine/threonine kinases (SnRK3), named CBL-interacting protein kinases (CIPK) [60]–[63]. For example, CBL1 and CBL9 were reported to interact and activate CIPK23 to enhance K^+^ uptake under low K^+^ condition and regulate leaf transpiration against drought stress in *Arabidopsis* [64], [65]. Another CIPK protein of CIPK7 was also interacted with CBL1 needed for clod stress tolerance [38]. Judging from the number of CBL-mediated stimuli identified so far and the target diversity exhibited by another sensor, CaM, it is possible that CBLs associate with a variety of proteins in addition to CIPKs to mediate more diverse signals. Actually, CBL1 has also been shown to interact with other proteins besides the CIPK proteins. Activity of another interactor of CBL1 named PI-4Kβ1 was mediated by CBL1, which was involved in growth of root hair [66]. A recent study revealed that CBL1 interacted and inactivated a specific PP2C-type phosphatase (PP2CA) for the activation of AKT1 channel with CIPK6 [67].

In this study, we confirmed that CBL1 physically interacted with AKINβ1, a regulatory β subunit of the SnRK1 complex that does not belong to the CIPK family. Based on previous studies, AtCBLs represents a multi-member protein family and the ‘specificity’ of calcium codes remains largely a mystery, raising an important question on the specificity of interaction with their targets. To assess specificity of AtCBL-AKINβ1 interaction and to identify other isoform-specific interactors, we conducted systematic yeast two-hybrid experiments to screen for AKINβ1 with different AtCBL isoforms. However, we did not find a physical interaction of AKINβ1 with any known CBLs except for CBL1 (data not shown), indicating a specific interaction between CBL1 and AKINβ1. SnRK1 belongs to a conserved family of protein kinases consisting of an α-catalytic subunit and regulatory β- and γ-subunits [68]. *Arabidopsis* AKINβ1, being such a SnRK1 subunit, is N-myristoylated and plays an important role in specificity of recognition between the SnRK1 complex and its targets [69]. Previous studies also showed that CBL1 possessed the conserved N-myristoylation motif and CBL1-GFP fusion proteins were observed on the plasma membrane of *Arabidopsis* protoplasts [70]. N-myristoylation is an important modification for membrane binding ability of cytoplasmic proteins [71]. Thus, it is possible that the two proteins form a complex attached to the plasma membrane as shown in Fig. 4C. It was originally identified that overproduction of *Arabidopsis* AKINβ1 caused developmental deficiency with less expanded cotyledons and no noticeable true leaves in the presence of glucose [27]. Thus, *cbl1* displays a glucose-sensitive phenotype similar to that observed in plants with *AKINβ1* overexpression. Hence CBL1 and AKINβ1 may exert opposite functions in sugar signaling. Interestingly, *SnRK1* overexpression also showed hypersensitivity to glucose and the capacity of SnRK1 to phosphorylate its targets was shown to be regulated by the availability of the non-catalytic AKINβ1 subunits [72], [73]. For example, a very recent study showed that the β regulatory subunit Gal83 was phosphorylated by the tomato AGC Ser/Thr protein kinase Adi3, leading to suppression of SnRK1 activity [74]. Furthermore, the CBL/CIPK complexes were able to phosphorylate target proteins to regulate signal transduction [75]. Considering all the previous reported results together with the evidence presented in this study, we propose that CBL1 together with certain CIPK or other protein kinases form a complex that affects the biological function of AKINβ1 through physical modification plus the interaction with AKINβ1, such as phosphorylation, then affecting the activity of SnRK1 in glucose response (Fig. 4D). However, such a CBL1-interacting protein kinase remains to be verified, and more evidence is needed for the biological relevance of the interaction between CBL1 and AKINβ1.

### GA Deficiency in *cbl1* Mutant Leads to Developmental Defective in the Presence of PAC

Phytohormones play important roles in regulation of developmental processes through close interaction with sugar signals. Previous studies showed that sugars stimulate ABA accumulation in seedling plants, and that ABA signal transduction is necessary for sugar-induced inhibition of photosynthesis and developmental arrest [76]. However, the present research demonstrated that *cbl1* did not affect its response to ABA as well as ethylene (Fig. 3A and B). This implies that CBL1 may be involved in sugar signaling by crosstalk with other phytohormones. GA is an important hormone for germination as shown by the observation that GA-deficient mutants do not germinate and germination can be induced in GA-deficient mutants by exogenous gibberellic acid (GA_3_) application [77]. Subsequent study showed that PAC severely repressed the germination of *cbl1* mutant compared with Col-0, whereas exogenous GA_3_ completely rescued the defect (Fig. 3C, D and E). Thus, CBL1 appears to affect the plant response to GA during seed germination and seedling growth. Further study revealed that expression of *GA3ox1* was significantly reduced in *cbl1* mutant plants (Fig. 3F). GAs are derived from geranylgeranyl diphosphate though a series reactions catalyzed by ent-copalyl diphosphate synthase (CPS) and ent-kaurene synthase (KS), GA 20-oxidase (AtGA20ox), and GA 3-oxidase (AtGA3ox) in higher plants [78]. *GA3ox1*, encoding GA 3-oxidase 1 and catalyzing the rate-limiting step of GA biosynthesis, is essential for GA synthesis during plant growth [79]. This suggests that repression of *CBL1* may lead to a deficiency in GA biosynthesis and signal response, and could be the reason why the *cbl1* mutant is hypersensitive to PAC (Fig. 3G). A previous report suggested that sugar and GA signals competed for tissue-specific regulation of α-amylase genes which are crucial in seed germination, indicating possible crosstalk between the sugar and GA signaling pathways [80]. And glucose has been reported to delay germination by repressing the GA signalling pathway via RGL2, which are negative regulators in GA signalling [14], [81]. Thus, we investigated whether glucose sensitivity was accompanied with GA deficiency in *cbl1*. Based on experiments with the exogenous application of hormones, hormone precursors, and hormone synthesis inhibitors, Dekkers et al. [6] concluded that glucose is not acting via the biosynthesis of GA. Next we found that seeds of *cbl1* mutant sown on glucose media or glucose media supplemented with 1 µM or 5 µM GA showed a similar germination curve (data not shown). Thus the germination-promoting GA was unable to counteract the negative effects of glucose and did not relieve the glucose inhibition in *cbl1*. To further study the glucose effects on the GA-signaling pathway, we analyzed the expression patterns of known GA-responsive genes such as *RGL2*, *SPY*, *GID1A* and *SLY1* [81]–[83]. According to the real-time PCR results, 3% glucose either differentially altered or not affected the expression of components of GA signaling as well as several genes involved in GA biosynthesis in Col-0 (Figure S3). However, these genes were also modified to a similar degree in *cbl1* mutant, suggesting that glucose causes no additional inhibition of GA signaling with repression of *CBL1*, and that sensitivity to glucose in *cbl1* may not be due to GA deficiency. Perhaps studies of more components of GA signaling under glucose treatment will contribute to a better understanding of the precise role of CBL1 in GA and glucose signal responses.

In conclusion, we show that suppression of *CBL1* affects plant responses to glucose and GA in *Arabidopsis*. The novel characteristics of CBL1 imply complex roles for the CBL protein family. Further investigation of the molecular mode of its action with interacting-proteins will be the focus of our future work to understand the role of CBL1 in sugar signaling.

## Supporting Information

Figure S1 **Nucleotide sequences of CBL1 promoter region and putative** ***cis*** **-acting elements.** The motifs with a significant similarity to the previously identified *cis*-acting elements are shown underline. The box indicates the translation initiation site. WBOXHVISO1: TGACT; WBBOXPCWRKY1: TTTGACY; SP8: AATAGTA; CMSRE-1 (Carbohydrate Metabolite Signal Responsive Element 1): TGGACGG; SURE: GAGAC; G motif: TACGTA; G box: CACGTG; “TATCCA” element: TATCCA; “A-box”: TATCCA; Pyrimidine box: CCTTTT.
(TIF)

Figure S2 **Effects of glucose and mannitol on seedling growth of wild-type (Col-0) and mutant (** ***cbl1*** **) plants.** Seven-day-old seedlings were transferred to vertical plates containing growth media supplemented with zero, 3% glucose or 3% mannitol. Photograph was taken after five days. Representative images were presented. Bars = 2 cm.
(TIF)

Figure S3 **Effect of glucose on GA biosynthetic and signaling pathway genes expression in Col-0 and** ***cbl1*** **mutant plants.** Seedlings were treated without or with 3% glucose for 6 h and harvested for RNA isolation. Relative amounts of each transcript were determined by real-time PCR and normalized relative to *UBQ10*. All values are averages of three independent experiments. *SLY1*, *SLEEP1*; *RGL2*, *DELLA protein*, *RGA-LIKE 2*; *SPY*, *SPINDLY*; *GID1A*, *GA INSENSITIVE DWARF1A*; *GA3ox*: *GA3-oxidase*.
(TIF)

Table S1 **Sequence information for primers used in real-time PCR.**
(DOC)
