# Supplementary figures and images for: A Novel Role for Arabidopsis CBL1 in Affecting Plant Responses to Glucose and Gibberellin during Germination and Seedling Development
Source: PLoS One. 2013 Feb 20;8(2):e56412. doi: 10.1371/journal.pone.0056412 (PMC3577912; doi:10.1371/journal.pone.0056412)

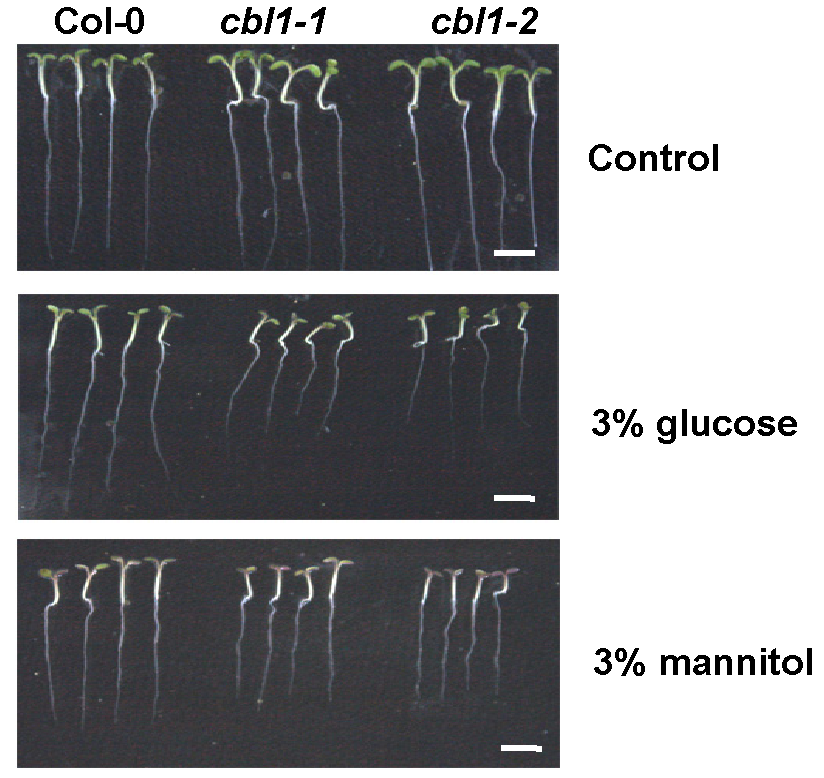

Supplement: Figure S2 — Effects of glucose and mannitol on seedling growth of wild-type (Col-0) and mutant ( cbl1 ) plants. Seven-day-old seedlings were transferred to vertical plates containing growth media supplemented with zero, 3% glucose or 3% mannitol. Photograph was taken after five days. Representative images were presented. Bars = 2 cm. (TIF) [file pone.0056412.s002.tif]
