# Supplementary material for: A Novel Role for Arabidopsis CBL1 in Affecting Plant Responses to Glucose and Gibberellin during Germination and Seedling Development
Source: PLoS One. 2013 Feb 20;8(2):e56412. doi: 10.1371/journal.pone.0056412 (PMC3577912; doi:10.1371/journal.pone.0056412)
Supplement: Table S1 — Sequence information for primers used in real-time PCR. (DOC) [file pone.0056412.s004.doc]

**Table 1.** Sequence information of the primers used for real-time PCR.

| Gene | Sequences | AGI number |
| --- | --- | --- |
| CBL1Forward | AATGAAACTGGCTGATGAAACC | AT4G17615 |
| CBL1Reverse | CCTCCGAATGGAAGACAAAACT |
| UBQ10Forward | GATCTTTGCCGGAAAACAATTGG | AT4G05320 |
| UBQ10Reverse | TAGAAAGAAAGAGATAACAGG |
| CAB1Forward | CACTGGTAAGGGACCGATAGAG | AT1G29930 |
| CAB1Reverse | ACACTCACGAAGCAAAGACTGA |
| APL3Forward | CTGTGGGTGTTTGAGGATGC | AT4G39210 |
| APL3Reverse | GATACCGCCATGTCAGAAGC |
| CHSForward | TCAAGCGTCTCATGATGTACC | AT5G13930 |
| CHSReverse | GTCGCCCTCATCTTCTCTTCC |
| ASN1Forward | AGGTGCGGACGAGATCTTTGG | AT3G47340 |
| ASN1Reverse | GTTGTCAATTGCCTTAAGTGG |
| PCForward | CCGTCAGCTCAAAACCTAAGAC | AT1G76100 |
| PCReverse | GACACCGAAATCCTTCAAAGAG |
| RBCSForward | GGCTAAGGAAGTTGACTACC | AT5G38410 |
| RBCSReverse | ACTTCCTTCAACACTTGAGC |
| G1D1A Forward | AAGAAAGCGGGTCAAGAGGT | AT3G05120 |
| G1D1A Reverse | ACAAACGCCGAAATCTCATC |
| GA1Forward | ACTCGTTGGAAGGTGTACCG | AT1G14920 |
| GA1Reverse | CTCAACTCGGTCAGGTCCAT |
| GA3ox1Forward | ACGTTGGTGACCTCTTCCAC | AT1G15550 |
| GA3ox1Reverse | CCCCAAAGGAATGCTACAGA |
| GA3ox2Forward | GGCGTAGCTCGTATTGCTTC | AT1G80340 |
| GA3ox2Reverse | GGAGAGCCAATAACGGTGAA |
| KSForward | CAATCGCAGCAAAGAAGTGA | AT1G79460 |
| KSReverse | TCTTTGCATTCCCTTGGAAC |
| SLY1Forward | ACGTCGACGCAAAGACCTTA | AT4G24210 |
| SLY1Reverse | GCAGCCGATGTTAGTCCAGT |
| RGL2Forward | GGCTGCACAGTGGAGGATTC | AT3G03450 |
| RGL2Reverse | CGCGCTAGATCCGAGATGA |
| SPYForward | GAGCTTGCTTTCCACTTTAATCCA | AT3G11540 |
| SPY2Reverse | ATCAAGGTTGTCACGGTCTTTGTA |
